# On the Management of Weak New-Born Infants

**Published:** 1867-04

**Authors:** 


### *On the management of Weak New-born Infants.* By professor DEPAUL.

(Journal of Practical Medicine and Surgery, October, 1866.)

Professor Depaul remarks that while abundant attention is given in obstetric treatises to the treatment of healthy new-born infants, and those who are seemingly still-born, little space is devoted to the care of the weakly. This want he endeavors in part to supply. He thinks that authors have not laid sufficient stress on certain deceptive appearances, which seem to imply that the infant is out of danger because it takes the breast, and seems to suck.

The fact is, however, one of very common occurrence; the infant apparently sucks, but does not increase in weight, and after a time discontinues its fruitless efforts, screams more frequent, and wastes away. In order to discover whether suction is efficiently performed, the child should at the time he appears to be taking the breast with most vigor, be removed from its nurse, and the presence or absence of milk in its mouth be ascertained. The paid nurses at the hospital are required every day to make this experiment. Mr.

Depaul also endeavors by all means to rouse from their indolence the wet-nurses to whom puny, delicate infants have been intrusted, when the nursling takes the breast but imperfectly. Under these circumstances, it often happens that the infant has not strength to suck, and the finest nurses are provided in vain. The best nurse in such cases, is not the woman who has the largest supply of milk, but one whose milk flows easily, and drops without effort into the child's mouth. If a nurse of this kind cannot be procured, milk of good quality should be obtained, and given mixed with thin gruel. Mr. Depaul agrees with Professor Scanzoni, that ass's milk is the best for the purpose; but in most cases the practitioner must be satisfied with cow's milk. Every hour or two, day and night, from one to three teaspoonfuls of diluted milk should be administered. Should this kind of food give rise to colic, Scanzoni recommends the addition of a little fennel or dill water; and as soon as the child has gained in strength, it is proper to procure for it a good wet-nurse; and this should not be too long delayed, lest the habit of receiving nutriment into its mouth without any effort, may prevent the infant ever taking to the breast again, a circumstance which occurred in the case of a young prince, at present living in exile; the nurse should then be instructed to draw her own milk with an exhausting glass; but this can seldom be obtained from a mercenary nurse, and scarcely ever succeeds but with mothers who rear their own children.

It should further be remarked, that in primiparæ the nipple is often so large or so hard, that if the child is not very strong, its efforts at suction are unavailing. The mother is then in fault, and it is therefore highly expedient to ascertain the condition of the breast in gravid women, in order to form an opinion as to the possibility of their nursing.

It is absolutely necessary, in addition to the measures calculated to restore and increase the strength of the infant, carefully to shield it from the influence of cold, and to adopt every precaution to preserve the temperature of the body at the physiological standard. Warmth is for infants, especially for new-born infants, the indespensable condition of the continuance of life. None but the strongest children can bear any loss of temperature. The weak invariably perish if exposed to cold; and Hunter sagaciously noted the fact, and strongly objected to the practice prevalent in his day of bathing very young children in cold water for the alleged purpose of invigorating their constitution. When, therefore, a child is prematurely born, or naturally weak, it should be carefully enveloped in warm clothing, kept in a comfortable bed, and guarded in every possible manner from adverse atmospherical influences. The thermometer should be daily consulted, and hot water bottles used, if necessary, to maintain the heat of the body at a proper height.

By means of these precautions, and if required by the exhibition of aromatic and stimulating remedies, Mr. Depaul has had the good fortune of restoring in the course of two or three weeks, children supposed not to be viable, to a normal state of development. Untiring supervision is always indispensable, as any neglect of these all-important points may entail irremediably fatal consequences.—*Half-Yearly Abstract.*

